# Meeting of the American Medical Association

**Published:** 1891-06

**Authors:** 


### Editorial.

### MEETING OF THE AMERICAN MEDICAL ASSOCIATION.

The recent meeting of the Association at Washington was a notable one in many particulars. And, while the meeting could not be called a truly representative one of the profession of this country, there were eminent men in attendance from all parts of the United States. The attendance was largely from the Western States, while from the East and South the attendance was very meager.

The entire representation, however, displayed abundant zeal for the advancement of the medical sciences and the elevation of the medical profession.

There is a determination on the part of the profession, as a body, to elevate the standard of medical education and to frown down and out of existence those medical colleges which do not conform to the standard prescribed by the Association. The colleges must extend the time necessary for graduation to three full courses of lectures and require greater proficiency on the part of applicants for degrees, or find their faculties and graduates debarred from the privileges of the American Medical Association and all other associations affiliating therewith.

The Journal extends its heartiest co-operation to the earnest workers in this important field. We hope to see this term of study upheld by all the colleges.

There seems also to be a very prevalent sentiment against the regulations prescribed by railroads, and other corporations, for the government of their surgeons. There is no more reason why a surgeon should do work for a railroad at greatly reduced prices than that a physician should engage to practice for wealthy, influential families at much lower fees than he charges the less favored. It is manifestly prejudicial to the interests of the profession and the public. We do not wish to be understood as casting any reflection upon railroad surgeons, for many of them are high-toned, honorable gentlemen who will readily acquiesce in the verdict of the profession.

The work in the various sections was of a high order and many valuable contributions to our general fund of information were made.

The social features were also very enjoyable, and few will there be who will not have pleasant recollections of the many elegant receptions, etc., tendered by the profession of Washington City.

### MUCH NEEDED LEGISLATION.

The laws regulating the practice of medicine in Georgia are sadly deficient in many particulars, and we hope the adjourned session of the legislature will amend them in several important respects.

There is the most urgent necessity for a State board of health and also a State licensing board. A bill ought to be introduced and passed providing for the establishment of both. There is no reason why such a bill might not be so framed as to treat all with perfect fairness. The public safety absolutely demands that something be done. The people are a ready prey to impostors and charlatans. Those who are in authority are in duty bound to protect the public from these pretenders, by putting in operation the necessary legal machinery.

We hope the bill now pending, requiring that all medical colleges shall demand that all applicants for graduation must have attended three full courses of lectures, will pass. Regulars, eclectics and homœopaths are agreed on that. Now, doctor, please, for the good of humanity as well as for that of our noble profession, appeal to your representatives and show them the necessity for such legislation and there will be no trouble about getting it. Something must be done to keep incompetent men from practicing upon the infirmities of the people.

### DR. GASTON AND THE ELASTIC LIGATURE.

At the recent meeting of the American Medical Association, in Washington City, Dr. Theo. A. McGraw, the Chairman of the section on surgery and anatomy, made an address upon the Elastic Ligature in the surgery of the intestines.

In the course of his remarks he referred to the original investigations of Dr. J. McF. Gaston, of this city, which appeared in this journal (1884).

We quote from Dr. McGraw's published paper (see *Journal of American Medical Association*, May 16):

"In 1884 Dr. J. McF. Gaston, of Atlanta, Ga., used it and other forms of ligatures in experiments on dogs which had for their purpose the establishment of a fistulous opening between the gall bladder and duodenum.

"I learned very recently from Professor Dr. Helfenrich, of Greifswald, that some experiments had been made by Dr. Franz Bardenheuer, at present an assistant in Professor von Bergmann's clinic in Berlin, which had for their object the production of intestinal anastomosis by the use of an elastic ligature, and through his great kindness have been enabled to secure a copy of Dr. Bardenheuer's paper from the author himself. . . . He, evidently ignorant of Dr. Gaston's previous experiments, operated once successfully on a dog by the same method, for the production of an anastomosis between the gall bladder and duodenum. These experiments of Dr. Bardenheuer do not seem, thus far, to have borne any practical fruit. Without knowing of any previous experiments with the elastic ligature, I began myself to experiment with it during the early part of the summer of 1890, hoping in this way to find a means of accomplishing the desired end of producing an anastomosis with previous adhesion of the intestinal surfaces.

"I had not, until this time, been aware that Dr. Gaston, of Atlanta, had anticipated me in this kind of work, but learned of the fact from Dr. Senn. Dr. Gaston kindly sent me the papers, excepting the first, which he had published on this subject, beginning in September, 1884. Dr. Gaston endeavored to establish fistulæ between the gall-bladder and duodenum by the use of a ligature, sometimes of silk and sometimes of rubber. The main features of his method were the same as those of my own. The gall-bladder was fastened by Lembert sutures to the intestine, the ligature passed through both viscera and tied, and other Lembert sutures again above and around the ligature. Many of his operations were complicated by the ligation of either the hepatic, cystic or common gall ducts, and in all of them, as nearly as I can judge from the description given, the amount of tissue included in the ligatures was much less than in my own experiments. His very interesting experiments were the first of their kind, and he can undoubtedly claim the priority in the attempts to make channels of communication between hollow viscera by means of ligatures, although I cannot learn that he ever attempted to do so for any other purpose than to make a duodeno-cholecystotomy.

---

DR. HOBART A. HARE, editor of the Philadelphia *Medical News*, has been elected Professor of Materia Medica and Therapeutics in the Jefferson Medical College, to succeed Dr. Roberts Bartholow. It is said that Dr. Hare will now resign his editorial duties.

DR. J. M. DACOSTA, who for twenty-seven years has been associated with the Jefferson Medical College, Philadelphia, has retired from the chair of Practice of Medicine.

A NEW era is dawning upon the history of medicine in the United States. Increased requirements have just been announced. Nine of the different States now require an attendance upon three complete courses of lectures and graduation, to entitle the candidate to practice medicine in their limits; and five of these are Southern States. All praise be to them! Other States are initiating measures for the accomplishment of the same end.—*Weekly Medical Review.*

It is proposed to open the medical department of the University of Texas in the autumn of this year. This department is to be at Galveston. The medical school will begin with nine professors and will give a three-years graded course of instruction of eight months each. Each of the professors will be paid, on an average, three thousand dollars per session. The Board of Regents will attempt to secure for the faculty comparatively young men, possessed of elements of success and capabilities of making a reputation for the institution.

The New York College of Physicians and Surgeons has been made a part of Columbia University. For many years the connection has existed practically in name only, but now the union is complete. The members of the medical faculty will draw their salary from the University fund. The University assumes entire financial control of the Medical College.

The Atlanta profession are much gratified to note the compliment which was paid their colleague, Dr. J. McF. Gaston, who was made Chairman of the Section on Surgery (for the session of 1892) at the recent meeting of the American Medical Association. Dr. Gaston presented a paper on Thoracic Surgery.

CASE OF EXTRA-UTERINE PREGNANCY.—J. W. Carhart, M. D. (*Texas Medical Journal*). In August, 1890, was called to see Mrs. V., 25 years of age, and on digital examination *per vaginam* found a tumor about as large as a hen's egg on the right side of the uterus, in the region of the right fallopian tube, some little lower than the tube, and seeming to involve the broad ligament. No fluctuation could be detected, no inflammation, but the lady complained of constant pain, extending at times down the right thigh. A distinct sulcus was clearly definable between the tumor and the fundus uteri, and the bladder appeared to be somewhat crowded, as she was troubled with frequent desire to micturate.

The battery was used with a light current, the negative pole in close proximity to the tumor, a glycero-cotton tampon placed in the vagina, and tincture of iodine applied externally. This treatment was continued daily for over a month, and after making a careful examination was convinced that it was a case of tubal pregnancy. As the patient was thin in flesh, relaxed, and somewhat anæmic the outlines of the tumor could readily be made out. The following day, after thorough antiseptic precautions, an aspirating needle was introduced into the tumor through the vault of the vagina, and after penetrating to the depth of about three quarters of an inch, resistance was distinctly felt. Some blood was withdrawn with no amniotic fluid, and it was thought that the needle had struck a multilocular cyst, passing through one sac and striking the walls of another.

A few weeks after this the patient suffered considerable pain for several hours, followed by quite a hæmorrhage. The next day in consultation the diagnosis of tubal pregnancy was made. The depression between the tube and uterus, though distinct, was lessened from first examination and the fundus uteri had enlarged somewhat, probably from the presence of blood clots.

A Barnes' bag was introduced into the womb with a silk cover to control dilatation, and with a surgical pump dilated to its full capacity. No anæsthetic was used, as the patient did not complain of pain. The next morning at an early hour, being hastily summoned to the lady, found a loop of the prolapsed cord protruding some three or four inches. The tumor had lessened and become one with the fundus. The neck of the womb was shortened, the canal patulous, and by a little effort the feet could be detected. The fœtus was delivered in a short time, followed quickly by the afterbirth, which was evidently in the tube and could be felt to contract, as the placenta passed out into the womb. This latter showed extensive fatty degeneration, but the fœtus was well formed, five and a half inches in length and of four months gestation. Recovery was slow but uneventful.

